# Social Roles, Subjective Age, and Gender: Exploring the Links in Later Life

**DOI:** 10.1093/geroni/igaa057.1828

**Published:** 2020-12-16

**Authors:** Anna Kornadt

**Affiliations:** University of Luxembourg, Esch-sur-Alzette, Diekirch, Luxembourg

## Abstract

Subjective age (SA) is strongly linked to positive developmental outcomes and successful aging. The social roles people assume are supposed to impact SA, since they incorporate age-graded social experiences and age-stereotypic role expectations. Social roles are also strongly gendered, providing the opportunity to understand gender-specific processes of SA. This study investigates a broad range of social roles and their relation to older men and women’s SA in later life. N = 285 participants aged 50 to 86 years (Mage = 65.04, SD = 8.88) reported on 19 social roles and their SA. Higher commitment to social roles of continued development and engagement was related to a younger subjective age, above and beyond sociodemographic variables, physical and mental health, but only for younger men. Commitment to family roles was related to a younger subjective age only for older men. Implications for the gender-specific understanding of antecedents of SA are discussed.

